# Changes in chemokine and growth factor levels may be useful biomarkers for monitoring disease severity in COVID-19 patients; a pilot study

**DOI:** 10.3389/fimmu.2023.1320362

**Published:** 2024-01-04

**Authors:** Blanka Wolszczak-Biedrzycka, Justyna Dorf, Marzena Wojewódzka-Żelezniakowicz, Małgorzata Żendzian-Piotrowska, Violetta Dymicka-Piekarska, Joanna Matowicka-Karna, Mateusz Maciejczyk

**Affiliations:** ^1^ Department of Psychology and Sociology of Health and Public Health, University of Warmia and Mazury in Olsztyn, Olsztyn, Poland; ^2^ Department of Clinical Laboratory Diagnostics, Medical University of Bialystok, Bialystok, Poland; ^3^ Department of Emergency Medicine and Disasters, Medical University of Bialystok, Bialystok, Poland; ^4^ Department of Hygiene, Epidemiology and Ergonomics, Medical University of Bialystok, Bialystok, Poland

**Keywords:** chemokines, growth factors, COVID-19, MEWS score, SARS-CoV-2

## Abstract

**Aim:**

The aim of the present study was to assess differences in the serum levels of chemokines and growth factors (GFs) between COVID-19 patients and healthy controls. The diagnostic utility of the analyzed proteins for monitoring the severity of the SARS-CoV- 2 infection based on the patients’ MEWS scores was also assessed.

**Materials and methods:**

The serum levels of chemokines and growth factors were analyzed in hospitalized COVID-19 patients (50 women, 50 men) with the use of the Bio-Plex Pro™ Human Cytokine Screening Panel (Biorad) and the Bio-Plex Multiplex system.

**Results:**

The study demonstrated that serum levels of MIP-1α, RANTES, Eotaxin, CTACK, GRO-α, IP-10, MIG, basic-FGF, HGF, SCGF-β, G-CSF, M-CSF, SCF, MIF, LIF, and TRAIL were significant higher in COVID-19 patients than in the control group. The concentrations of CTACK, GRO-α, IP-10, MIG, basic-FGF, HGF, PDGF- BB, GM-CSF, SCF, LIF, and TRAIL were higher in asymptomatic/mildly symptomatic COVID-19 patients (stage 1) and COVID-19 patients with pneumonia without respiratory failure (stage 2). The receiver operating characteristic (ROC) analysis revealed that IP-10, MIF, MIG, and basic-FGF differentiated patients with COVID-19 from healthy controls with the highest sensitivity and specificity, whereas GM-CSF, basic-FGF, and MIG differentiated asymptomatic/mildly symptomatic COVID-19 patients (stage 1) from COVID-19 patients with pneumonia without respiratory failure (stage 2) with the highest sensitivity and specificity.

**Conclusions:**

MIG, basic-FGF, and GM-CSF can be useful biomarkers for monitoring disease severity in patients with COVID-19.

## Introduction

The cytokine release syndrome (CRS), namely excessive cytokine generation caused by the migration of immune cells to the site of inflammation, is one of the key mechanisms responsible for the development of COVID-19 symptoms (1). In patients infected with the SARS-CoV-2 virus, the main mediators of inflammation include the tumor necrosis factor- alpha (TNF-α), interleukin 1β (IL-1β), and interleukin 6 (IL-6) (2–4). However, chemokines and growth factors (GFs) also significantly contribute to CRS in the progression of COVID-19 (5).

The following chemokines play the most important role in COVID-19 progression: monocyte chemoattractant protein-1 (MCP-1/CCL2), macrophage inflammatory protein-1 alpha (MIP-1 α/CCL3), macrophage inflammatory protein 1-beta (MIP-1 β/CCL4), regulated upon activation, normal T-cell expressed and secreted (RANTES/CCL5), monokine induced by γ-interferon (MIG/CXCL9), and interferon-inducible protein (IP-10/CXCL10) (6–8). Chemokines are chemotactic cytokines that are secreted by monocytes, macrophages, circulating blood dendritic cells, fibroblasts, granulocytes, and epithelial cells in response to pathogens, including SARS-CoV-2 virions (9). Chemokines are expressed in inflamed tissues; they affect the maturation and differentiation of immune cells, mainly T cells, neutrophils, eosinophils, and macrophages, and they stimulate the migration of immune cells to the inflammation site (10). Chemokines and GFs not only fight inflammation, but also participate in the process of healing damaged tissues in COVID-19 patients (9). The hepatocyte growth factor (HGF) regulates the proliferation of hepatocytes and skeletal muscle cells (11). Similarly to chemokines, colony-stimulating factors (CSFs) produced by lymphocytes, endothelial cells, fibroblasts, and epithelial cells influence the populations of granulocytes and macrophages, and mobilize these cells to fight the infection and prevent the acute respiratory distress syndrome (12).

Similarly to other cytokines (IL-6, TNFα), changes in the concentrations of chemotactic cytokines and GFs can be potentially useful diagnostic or prognostic markers and promising therapeutic targets for monitoring COVID-19 progression. Some chemokines, including MCP- 1, RANTES, and the liver and activation-regulated chemokine (LARC) are regarded as novel biomarkers in inflammatory skin conditions, advanced atherosclerosis, and diabetes (13, 14). The stromal cell-derived factor 1 (SDF-1/CXCL12) chemokine has been also examined as a potential target for therapeutic intervention in patients with colorectal, breast, and lung cancer (15–17).

From the clinical point of view, changes in the serum levels of chemokines and GFs could provide valuable information about disease progression in patients infected with the SARS-CoV-2 virus. To date, most researchers have analyzed chemokine and GF concentrations in the blood serum of patients in the acute phase of COVID-19 (18, 19), and this is the first study to evaluate changes in chemokine and GF levels in asymptomatic/mildly symptomatic patients, as well as in patients with pneumonia without respiratory failure. The presented results not only expand the existing knowledge on immune processes in these groups of patients, but they can also be used as prognostic biomarkers for monitoring disease severity in patients with COVID-19, and as potential therapeutic targets.

## Materials and methods

### Description of the study

The study was approved by the Bioethics Committee of the Medical University in Białystok (decision No. APK.002.353.2021). All research participants gave their written consent to participate in the study.

### Study group

The study group consisted of 100 patients with a positive result of a COVID-19 PCR test (nasopharyngeal swab) who were admitted to the Emergency Department of the University Clinical Hospital in Białystok between 20 January and 20 November 2021. Blood samples were collected from patients who tested positive for COVID-19 in the PCR test which was administered upon admission to the Emergency Department.

The severity of COVID-19 was assessed based on the Modified Early Warning Score (MEWS) (20) which is recommended by the Polish Society of Epidemiology and Infectious Diseases and relies on the following parameters: systolic blood pressure, heart rate, respiratory rate, body temperature, and neurological symptoms. Four stages of COVID-19 progression were described based on the above parameters: 1) asymptomatic and mildly symptomatic infection, 2) symptomatic infection with pneumonia without symptoms of respiratory failure, 3) symptomatic infection with pneumonia and symptoms of respiratory failure, 4) symptomatic infection with multiple organ failure (**Table 1**).

**Table 1:** Modified Early Warning Score (MEWS).

Score	1	2	3	4
Respiratory rate, breaths/min	9-14	15-20	21-29 or ≤8	>29
Heart rate, bpm	51-100	101-110 or 41-50	111-129 or ≤40	>129
Systolic blood pressure, mm Hg	101-199	81-100	≤200 or 71-80	≤70
Hourly urine, mL/kg of body weight/h	>0.5		<0.5	Nil
Body temperature, °C	36.1-38	38.1-38.5 or 35.1-36	≤38.6 or ≤35	
Neurological symptoms	Alert	Responsive to voice	Responsive to pain	Unresponsive

The study group was divided into two subgroups. Subgroup 1 consisted of asymptomatic and mildly symptomatic patients (MEWS 1), whereas subgroup 2 consisted of symptomatic patients with pneumonia without symptoms of respiratory failure (MEWS 2). None of the patients had symptoms characteristic of stage 3 and 4 COVID-19 progression based on their MEWS scores (9).

Demographic parameters (sex, age), length of hospital stay (days), comorbidities (present, absent), hematological disorders (present, absent), diabetes (present, absent), hypertension (present, absent), obesity (present, absent), heart disease (present, absent), history of cancer (present, absent), and clinical symptoms, including fever (present, absent), cough (present, absent), dyspnea (present, absent) and the acute respiratory distress syndrome (ARDS) (present, absent), were analyzed. The patients were subjected to imaging examinations (radiography and computed tomography of the chest) and laboratory tests, including complete blood count (CBC), coagulation parameters (PT, APTT, D-dimers), kidney function tests (creatinine levels with estimated glomerular filtration rate (eGFR), urea), electrolyte levels (Na^+^, K^+^), and lactate dehydrogenase (LDH) activity.

### Control group

The control group consisted of 50 healthy subjects who performed routine employee tests in a laboratory in Bialystok (Poland) and tested negative for COVID-19.

### Materials

In both groups, blood for analyses was collected from the basilic vein into clot activator tubes. The serum was separated by centrifugation (1000 × g, 20 minutes), and the samples were stored at a temperature of -80°C until analysis.

### Chemokine and GF detection

The serum levels of chemokines and GFs in control group and study group patients were determined with the use of the Bio-Plex Pro™ Human Cytokine Screening Panel (Biorad) and the Bio-Plex Multiplex system based on the Luminex xMAP technology. The concentrations of the following chemokines were analyzed: MCP-1, MIP-1α, MIP-1β, RANTES, eotaxin, cutaneous T cell-attracting chemokine (CTACK), growth-regulated oncogene-alpha (GRO-α), interferon gamma-induced protein (IP-10), and MIG. The analyzed GFs were: basic fibroblast growth factor (basic-FGF), hepatocyte growth factor (HGF), platelet-derived growth factor (PDGF-BB), stem cell growth factor-beta (SCGF-β), granulocyte colony-stimulating factor (G- CSF), granulocyte-macrophage colony-stimulating factor (GM-CSF), macrophage colony- stimulating factor (M-CSF), stem cell factor (SCF), macrophage migration inhibitory factor (MIF), leukemia inhibitory factor (LIF), stromal cell-derived factor (SDF-1α), tumor necrosis factor-related apoptosis-inducing ligand (TRAIL), and interferon gamma (INF-γ). All assays were performed in duplicate serum samples.

### Statistical analysis

Statistical analyses were conducted with the use of GraphPad Prism 9.0 software (GraphPad Software, La Jolla, USA). The Shapiro–Wilk test was used to determine the normality of distribution. The Student’s t-test was applied to test data with a normal distribution, and the Mann-Whitney test was used to analyze data that did not follow a normal distribution. The results were presented as the median (minimum-maximum) at a significance level of p<0.05.

## Results

### Characteristics of the study group

The study group consisted of 100 patients infected with the SARS-CoV-2 virus, aged 36 to 87 years (65 females and 35 males). The patients were divided into two subgroups based on their MEWS scores (**Table 1**): stage 1 (asymptomatic/mildly symptomatic) – 53 subjects, and stage 2 (pneumonia without respiratory failure) – 47 subjects. Fifty-seven of the evaluated patients reported comorbidities, including hypertension (36 patients), coronary artery disease (26 patients), and diabetes (17 patients). The most prevalent symptoms were fever and dyspnea which were noted in 37 and 35 patients, respectively. The hospital length of stay was less than 10 days in 67 patients, 10-20 days in 12 patients, and more than 20 days in 21 patients. The studied population is characterized in **Table S1**, and the results of laboratory tests are shown in **Table S2**.

### Serum levels of chemokines and GFs in patients infected with the SARS-CoV-2 virus relative to the control group

**Figure 1:**
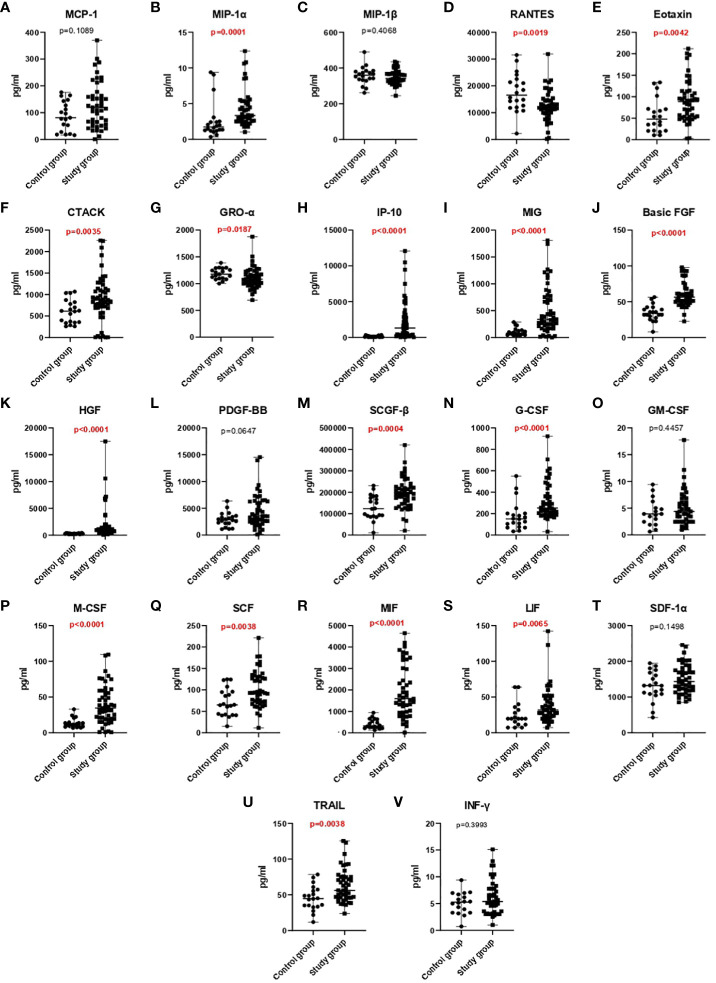
A comparison of chemokine **(A-I)** and growth factor **(J-U)** and INF-γ **(V)** concentrations in the control group and the study group.

The serum levels of most chemokines and GFs, excluding MIP-1β, RANTES, GRO-α, GM, and INF- γ, were higher in COVID-19 patients than in healthy controls. Significant differences were noted in the values of the following parameters: MIP-1α (p=0.0001), RANTES (p=0.0019), Eotaxin (p=0.0042), CTACK (p=0.0035), GRO-α (p=0.0187), IP-10 (p<0.0001), MIG (p<0.0001), basic-FGF (p<0.0001), HGF (p<0.0001), SCGF-β (p=0.0004), G-CSF (p<0.0001), M-CSF (p<0.0001), SCF (p=0.0038), MIF (p<0.0001), LIF (p=0.0065), and TRAIL (p=0.0038) (**Figure 1**, **Table 2**).

**Table 2:** Serum levels of chemokines and growth factors in COVID-19 patients and the control group.

Parameter	Control group	COVID-19 patients	p-value
	Median (min-max) (pg/mL)	Median (min-max) (pg/mL)	
**MCP-1**	81.28 (16.22-176.2)	119.5 (1.000-369.8)	0.1089
**MIP-1α**	1.735 (0.3500-9.380)	3.33 (1.000-12.36)	0.0001
**MIP-1β**	360 (261.3-489.2)	341.2 (244.8-435.1)	0.4068
**RANTES**	16538 (2220-31531)	11868 (221.0-31886)	0.0019
**Eotaxin**	47.71 (10.46-133.5)	88.57 (2.740-212.1)	0.0042
**CTACK**	613.5 (258.5-1068)	873.3 (2.610-2260)	0.0035
**GRO-α**	1176 (1001-1390)	1084(693.2-1878)	0.0187
**IP-10**	93.09 (13.53-342.7)	1301 (18.17-12086)	<0.0001
**MIG**	78.58 (33.39-285.9)	337 (1.000-1810)	<0.0001
**Basic-FGF**	33.64 (7.910-56.42)	56.42 (22.61-98.01)	<0.0001
**HGF**	289.2 (155.4-433.4)	951 (72.59-17473)	<0.0001
**PDGF-BB**	2854 (1101-6347)	3557 (110.9-14546)	0.0647
**SCGF-β**	122865 (11003-231021)	193277 (20574-419873)	0.0004
**G-CSF**	150.4 (34.62-551.6)	252.1 (31.60-922.1)	<0.0001
**GM-CSF**	3.92 (0.6200-9.430)	4.37 (0.9800-17.72)	0.4457
**M-CSF**	11.41 (6.770-32.96)	34.54 (0.8700-109.4)	<0.0001
**SCF**	64.83 (15.36-124.7)	93.04 (11.54-221.1)	0.0038
**MIF**	325.1 (126.4-940.5)	1596 (14.79-4648)	<0.0001
**LIF**	19.69 (7.270-63.88)	31.89 (7.270-142.2)	0.0065
**SDF-1α**	1319 (427.6-1947)	1434 (853.2-2456)	0.1498
**TRAIL**	44.69 (11.94-78.39)	56.21 (23.65-125.3)	0.0038
**INF-γ**	5.25 (0.7300-9.380)	5.38 (1.000-15.12)	0.3993

(MCP-1), monocyte chemoattractant protein 1; (MIP-1 α, MIP-1 β), macrophage inflammatory protein-1 α and β; (RANTES), regulated on activation, normal T-cell expressed and secreted; eotaxin, (CTACK), cutaneous T cell-attracting chemokine; (GRO-α), growth- regulated oncogene-α; (IP-10), interferon gamma-induced protein; (MIG), monokine induced by interferon-γ; (basic-FGF), basic fibroblast growth factor; (HGF), hepatocyte growth factor; (PDGF-BB), platelet-derived growth factor; (SCGF-β), stem cell growth factor-beta; (G-CSF), granulocyte colony-stimulating factor; (GM-CSF), granulocyte-macrophage colony-stimulating factor; (M-CSF), macrophage colony-stimulating factor; (SCF), stem cell factor; (MIF), macrophage migration inhibitory factor; (LIF), leukemia inhibitory factor; (SDF-1α), stromal cell- derived factor; (TRAIL), tumor necrosis factor-related apoptosis-inducing ligand and (INF-γ), interferon gamma.

### ROC analysis of chemokines and GFs in COVID-19 patients and healthy controls

**Figure 2:**
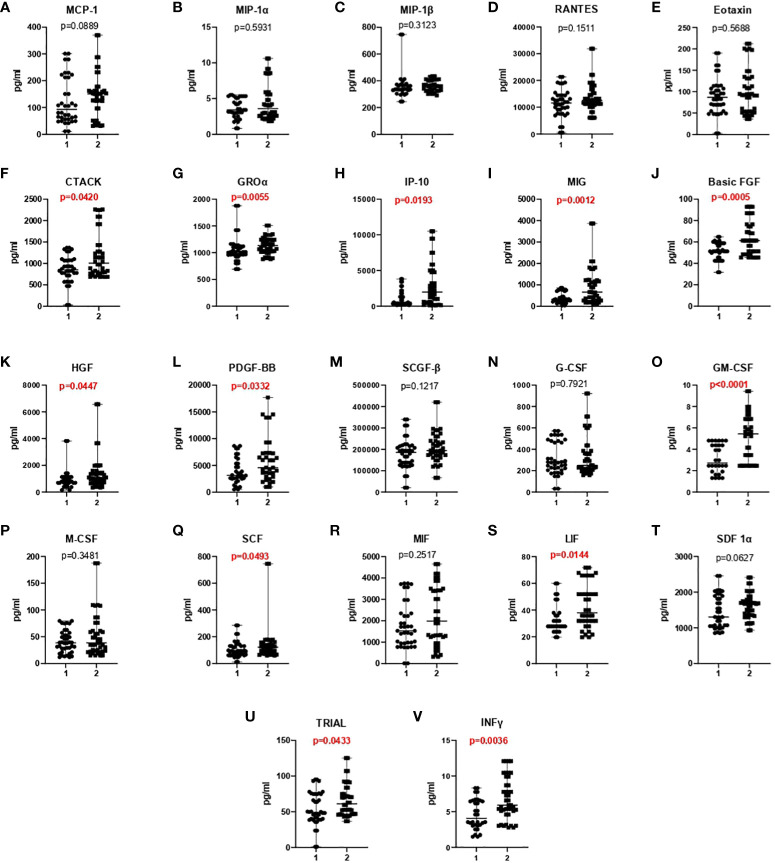
A comparison of chemokine **(A-I)**, growth factor **(J-U)**, and INF-γ **(V)** concentrations in asymptomatic/mildly symptomatic COVID-19 patients (stage 1) and COVID-19 patients with pneumonia without respiratory failure (stage 2).

**Figure 3:**
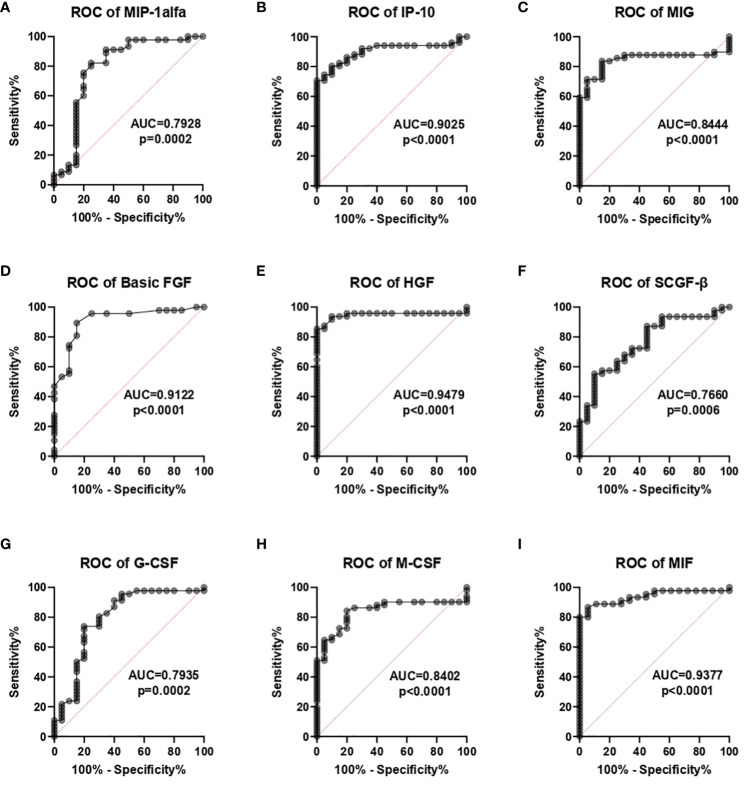
Receiver operating characteristic (ROC) analysis of chemokines **(A-C)** and growth factors **(D-I)** in COVID-19 patients and healthy controls.

The receiver operating characteristic (ROC) analysis demonstrated that the following chemokines: MIP-1α, IP-10, and MIG, and the following GFs: basic-FGF, HGF, SCGF-β, G- CSF, M-CSF, and MIF can be useful parameters for diagnosing COVID-19 patients (**Table 3**). The optimal cut-off values were calculated in the ROC analysis, and ROC curves are presented in **Figure 2**. The area under the curve (AUC) for MIP-1 α, IP-10, MIG, basic-FGF, HGF, SCGF- β, G-and MIF was determined at 0.9633, 0.8602, 0.9864, 0.9642, 0.9304, 0.9468 and 0.8942, respectively. The optimal cut-off values for MIP-1α, IP-10, MIG, basic-FGF, HGF, SCGF-β, G-CSF, M-CSF, and MIF were determined at 2.535pg/mL, 243.8 pg/mL, 160.8 pg/mL, 3.91 pg/mL, 372.1 pg/mL, 158340 pg/mL, 202.9 pg/mL, 14.91 pg/mL, and 707.3 pg/mL, respectively (**Figure 3**, **Table 3**).

**Table 3:** Area under the curve (AUC) values of chemokines and growth factors that were used to differentiate between COVID-19 patients and the control group.

Parameter	AUC	p-value	Cut-off	Sensitivity (%)	Specificity (%)	95% confidence interval
**MIP-1 α**	0.7928	0.0002	2.535	75.56	80	0.6495 to 0.9361
**IP-10**	0.9025	<0.0001	243.8	82.35	85	0.8311 to 0.9738
**MIG**	0.8444	<0.0001	160.8	83.67	85	0.7491 to 0.9397
**Basic-FGF**	0.9122	<0.0001	3.91	89.36	85	0.8357 to 0.9888
**HGF**	0.9479	<0.0001	372.1	93.75	90	0.8897 to 1.000
**SCGF-β**	0.766	0.0006	158340	72.34	65	0.6455 to 0.8864
**G-CSF**	0.7935	0.0002	202.9	73.91	80	0.6600 to 0.9270
**M-CSF**	0.8402	<0.0001	14.91	84.31	80	0.7458 to 0.9346
**MIF**	0.9377	<0.0001	707.3	88.89	88.89	0.8789 to 0.9964

(MIP-1 α), macrophage inflammatory protein-1 α; (IP-10), interferon gamma-induced protein; (MIG), monokine induced by interferon-γ; (basic- FGF), basic fibroblast growth factor; (HGF), hepatocyte growth factor; (SCGF-β), stem cell growth factor-beta; (G-CSF), granulocyte colony-stimulating factor; (M-CSF), macrophage colony-stimulating factor; (SCF), stem cell factor; (MIF) macrophage migration inhibitory factor.

### Serum levels of chemokines and GFs in asymptomatic/mildly symptomatic COVID-19 patients (stage 1) and COVID-19 patients with pneumonia without respiratory failure (stage 2)

An analysis of chemokine and GF concentrations revealed that the serum levels of all evaluated proteins, excluding M-CSF, were higher in COVID-19 patients with pneumonia without respiratory failure than in asymptomatic/mildly symptomatic COVID-19 patients. Significant differences (p<0.05) were noted in the values of the following parameters: CTACK (p=0.0420), GRO-α (p=0.0055), IP-10 (p=0.0193), MIG (p=0.0012, basic-FGF (p=0.0005), HGF (p=0.0447), PDGF-BB (p=0.0332), GM-CSF (p<0.0001), SCF (p=0.0493), LIF (p=0.0144), TRAIL (p=0.0433), and INF-γ (p=0.0036) (**Figure 2**, **Table 4**).

**Table 4:** Serum levels of chemokines and growth factors in asymptomatic/mildly symptomatic COVID-19 patients (stage 1) and COVID-19 patients with pneumonia without respiratory failure (stage 2).

Parameter	MEWS 1	MEWS 2	*p-value*
	Median (min-max) (pg/ml)	Median (min-max) (pg/ml)	
**MCP-1**	92.76 (11.57-301.2)	150.7 (31.44-369.8)	0.0889
**MIP-1α**	3.410 (0.8400-5.490)	3.575 (1.860-10.61)	0.5931
**MIP-1β**	338.2 (244.8-746.6)	359.9 (292.0-435.1)	0.3123
**RANTES**	11596 (534.2-21335)	12542 (6079-31886)	0.1511
**Eotaxin**	86.83 (2.740-190.0)	92.91 (36.36-212.1)	0.5688
**CTACK**	850.2 (27.92-1368)	1007 (684.7-2260)	0.0420
**GRO-α**	1013 (693.2-1878)	1132 (878.0-1507)	0.0055
**IP-10**	545.1 (133.3-3818)	1979 (123.1-10489)	0.0193
**MIG**	328.6 (39.22-863.6)	665.6 (111.6-3865)	0.0012
**Basic-FGF**	51.17 (31.72-64.78)	61.31 (45.45-92.99)	0.0005
**HGF**	760.1 (152.7-3822)	1031 (341.6-6569)	0.0447
**PDGF-BB**	3152 (507.2-8640)	4582 (992.5-17685)	0.0332
**SCGF-β**	185801 (20574-339542)	194055 (66258-419873)	0.1217
**G-CSF**	275.8 (31.60-571.9)	249.4 (158.5-922.1)	0.7921
**GM-CSF**	2.715 (1.310-4.810)	5.440 (2.460-9.430)	<0.0001
**M-CSF**	38.60 (11.99-79.52)	38.50 (15.30-187.2)	0.3481
**SCF**	92.14 (11.54-285.2)	122.0 (60.38-745.8)	0.0493
**MIF**	1532 (14.79-3748)	1985 (316.7-4648)	0.2517
**LIF**	27.84 (19.69-59.91)	37.94 (19.69-71.80)	0.0144
**SDF-1α**	1304 (853.2-2456)	1670 (931.4-2412)	0.0627
**TRAIL**	49.12 (1.000-95.21)	61.22 (36.82-125.3)	0.0433
**INF-γ**	4.070 (1.490-8.310)	5.910 (2.770-12.10)	0.0036

(MCP-1), monocyte chemoattractant protein 1; (MIP-1 α, MIP-1 β), macrophage inflammatory protein-1 α and β; (RANTES), regulated on activation, normal T-cell expressed and secreted eotaxin; (CTACK), cutaneous T cell-attracting chemokine; (GRO-α), growth- regulated oncogene-α (IP-10), interferon gamma-induced protein; (MIG), monokine induced by interferon-γ; (basic-FGF), basic fibroblast growth factor; (HGF), hepatocyte growth factor; (PDGF-BB), platelet-derived growth factor; (SCGF-β), stem cell growth factor-beta; (G-CSF), granulocyte colony-stimulating factor; (GM-CSF), granulocyte-macrophage colony-stimulating factor; (M-CSF), macrophage colony-stimulating factor; (SCF), stem cell factor; (MIF), macrophage migration inhibitory factor; (LIF), leukemia inhibitory factor; (SDF-1α), stromal cell- derived factor; (TRAIL), tumor necrosis factor-related apoptosis-inducing ligand and (INF-γ), interferon gamma.

### ROC analysis of chemokines and GFs in asymptomatic/mildly symptomatic COVID-19 patients (stage 1) and COVID-19 patients with pneumonia without respiratory failure (stage 2).

**Figure 4:**
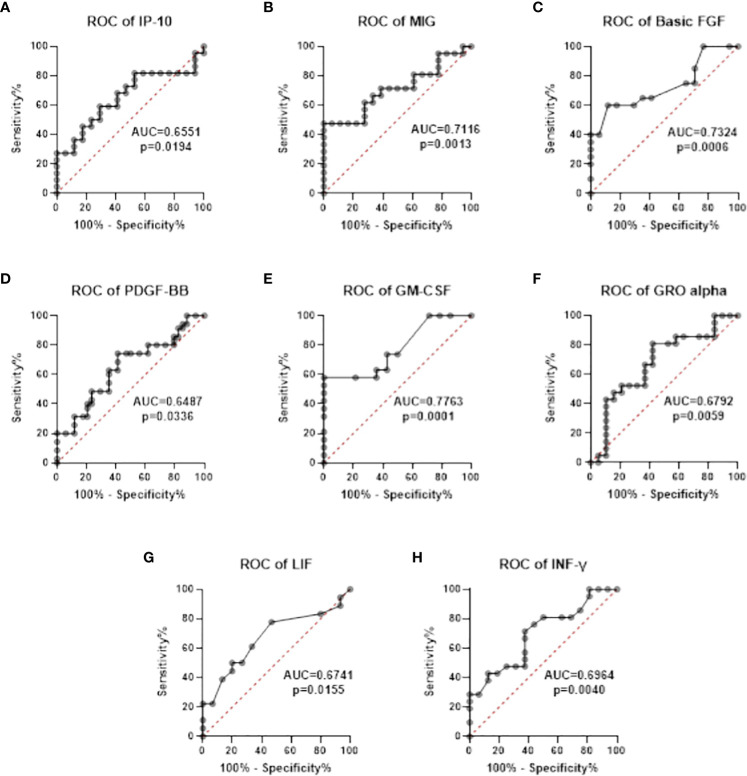
Receiver operating characteristic (ROC) curves of chemokines **(A, B)** and growth factors **(C-G)** and INF-γ **(H)** that were used to differentiate between asymptomatic/mildly symptomatic COVID-19 (stage 1) and COVID-19 patients with pneumonia without respiratory failure (stage 2).

The ROC analysis revealed that chemokines: IP-10 and MIG, and GFs: basic-FGF, PDGF-BB, GM-CSF, GRO-α, LIF, and INF-γ can be useful parameters for differentiating between asymptomatic/mildly symptomatic COVID-19 patients and patients with pneumonia without respiratory failure (**Table 5**). The optimal cut-off values were calculated in the ROC analysis, and ROC curves are presented in **Figure 4**. The AUC values of IP-10, MIG, basic-FGF, PDGF- BB, GM-CSF, GRO-α, LIF, and INF-γ were determined at 0.6551, 0.7116, 0.7324, 0.6487, 0.7763, 0.6792, 0.6741, and 0.6964, respectively. The optimal cut-off values for IP-10, MIG, basic-FGF, PDGF-BB, GM-CSF, GRO-α, LIF, and INF-γ were determined at 1290, 410.1, 54.47, 3945, 4.035, 1054, 33.91, and 5.315, respectively (**Figure 4**, **Table 5**).

**Table 5:** Area under the curve (AUC) values of chemokines and growth factors that were used to differentiate between asymptomatic/mildly symptomatic COVID-19 (stage 1) and COVID- 19 patients with pneumonia without respiratory failure (stage 2).

Parameter	AUC	p-value	Cut-off	Sensitivity (%)	Specificity (%)	95% confidence interval
**IP-10**	0.6551	0.0194	1290	59.09	64.71	0.5331 to 0.7771
**MIG**	0.7116	0.0013	410.1	66.67	66.67	0.5969 to 0.8264
**Basic-FGF**	0.7324	0.0006	54.47	65.00	64.71	0.6180 to 0.8468
**PDGF-BB**	0.6487	0.0336	3945	62.86	64.71	0.5185 to 0.7790
**GM-CSF**	0.7763	0.0001	4.035	66.16	64.29	0.6657 to 0.8869
**GRO-alpha**	0.6792	0.0059	1054	66.67	63.16	0.5586 to 0.7998
**LIF**	0.6741	0.0155	33.91	61.11	66.67	0.5434 to 0.8048
**INF-γ**	0.6964	0.0040	5.315	66.67	62.50	0.5772 to 0.8157

(IP-10), interferon gamma-induced protein; (MIG), monokine induced by interferon-γ (basic-FGF), basic fibroblast growth factor; (PDGF-BB), platelet-derived growth factor; (GM-CSF), granulocyte-macrophage colony-stimulating factor; (GRO-α), growth-regulated oncogene-α; (SCF), stem cell factor; (LIF), leukemia inhibitory factor; (INF-γ) interferon gamma.

## Discussion

The search for new biomarkers supporting assessments of the severity of COVID-19 is very important. It should be noted that the condition of patients infected with the SARS-CoV-2 virus can deteriorate rapidly (21–23). Therefore, a fast and accurate diagnosis based on clinical symptoms and the results of laboratory tests, followed by patient monitoring, play a key role in detecting deterioration in vital signs. The immune response to infection with the SARS-CoV-2 virus is a complex process that involves many cell populations and humoral factors (24, 25), but cytokines (26), including chemokines and GFs (27), are the key inflammation-regulating factors. Chemokines are peptides comprising 70-130 amino acids (28), and they have been divided into four subfamilies based on their chemical structure: CXC (alpha) chemokines, CC (beta) chemokines, C (gamma) chemokines, and CX3C (delta) chemokines, where C denotes cysteine residues and X denotes amino acid residues (29). Until recently, scientists assumed that chemokines are responsible only for stimulating the migration of leukocytes from the blood to tissues and inflammation foci (30). However, recent research has shown that chemokines also affect other processes by stimulating leukocyte chemotaxis, regulating intracellular processes and immune responses, and participating in the pathogenesis of inflammatory (31–33), autoimmune and proliferative diseases (34). Chemokines also influence the activation of adhesive molecules, the activation and differentiation of leukocytes, and they regulate cell proliferation (35).

**Figure 5:**
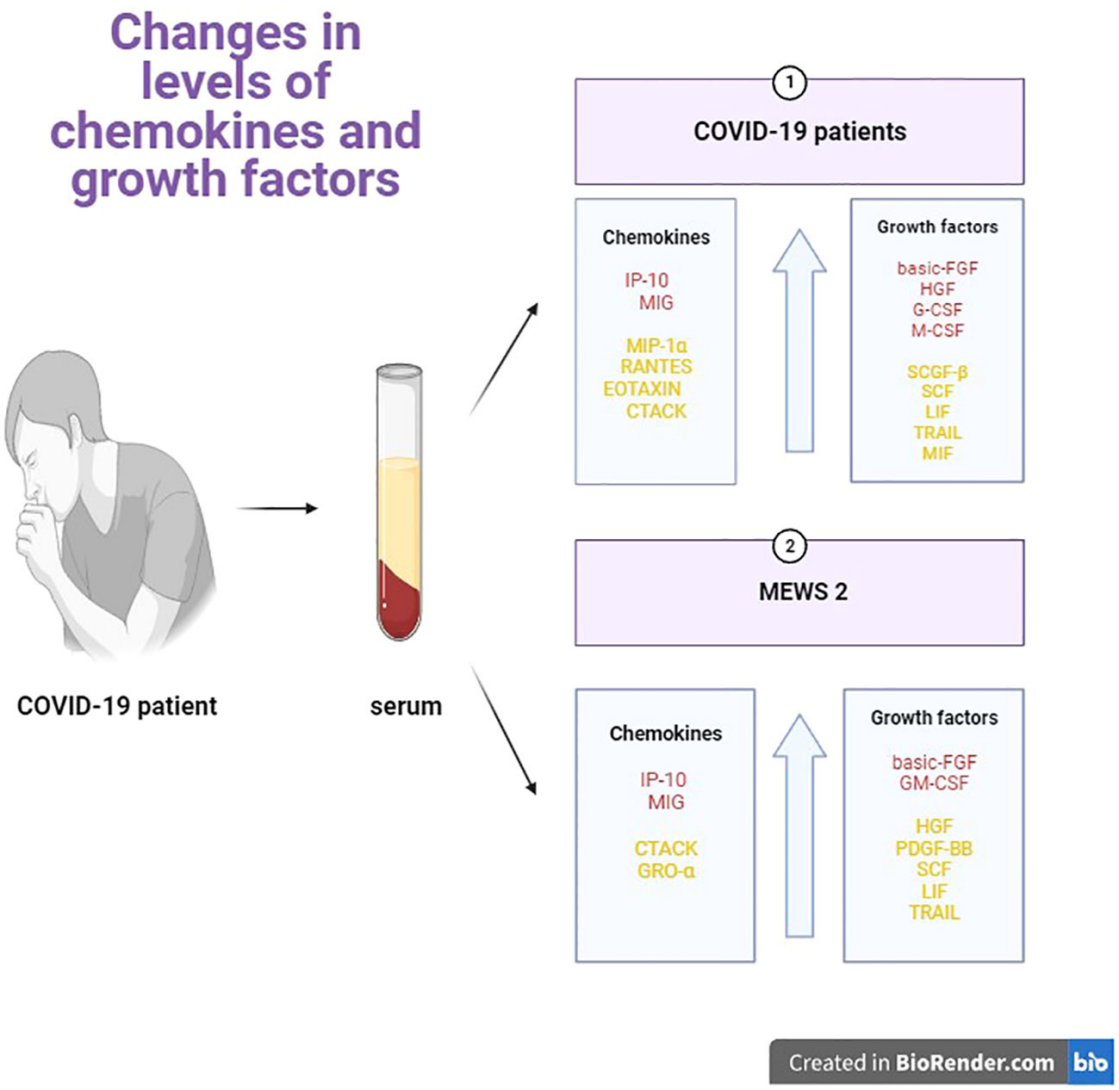
Graphical comparison of the serum levels of chemokines and growth factors in patients with COVID-19 vs healthy controls, and in patients with stage 2 vs stage 1 COVID-19.

In the present study, the serum levels of most of the analyzed chemokines and growth factors (excluding MCP-1, MIP-1β, RANTES, and GRO-α) were significantly higher (p<0.05) in COVID-19 patients than in the control group. In the current study, these parameters were compared in patients in the early stages of COVID-19 (MEWS 1 vs. MEWS 2). Symptomscharacteristic of stage 1 (asymptomatic/mildly symptomatic) and stage 2 (pneumonia without respiratory failure) COVID-19 progression were identified based on the patients’ MEWS scores. The serum levels of nearly all analyzed chemokines and growth factors (excluding MIP-1β) were higher in stage 2 than stage 1 patients (**Figure 5**), which suggests that the analyzed chemokines, in particular GM-CSF, basic-FGF and MIG, have high diagnostic value (AUC of 0.7116, 0.7324 and 0.7763, respectively) and can be used as effective markers for monitoring disease severity in COVID-19 patients.

MIG and IP-10 were the most effective diagnostic markers for differentiating between asymptomatic/mildly symptomatic COVID-19 patients and COVID-19 patients with pneumonia without respiratory failure. Both chemokines belong to the CX subfamily and the non-ELR subgroup (36, 37). Non-ELR chemokines lack the ELR (Glu-Leu-Arg) motif, and they act as chemoattractants and activators of monocytes, dendritic cells, lymphocytes (T, B, and NK cells), basophils and eosinophils, without angiogenic potential (38, 39). IP-10 and MIG bind to chemokine receptor 3 (CXCR3), and they play a particularly important role in the Th14 immune response by recruiting leukocytes to the site of inflammation (40). In the present study, the increase in IP-10 and MIG levels in stage 2 COVID-19 patients could point to immune system activation and a stronger inflammatory response, which increases the risk of serious complications (organ failure) in stage 2 than in stage 1 patients. To date, most studies have evaluated the serum levels of IP-10 and MIG in advanced stages of COVID-19 characterized by respiratory failure and/or multiple organ failure (41, 42). The concentration of IP-10 was significantly higher in COVID-19 patients with acute pneumonia than moderate pneumonia (43). In addition, the serum levels of IP-10 were considerably higher in patients with the acute respiratory distress syndrome (ARDS) and patients who died from the complications associated with the SARS-CoV-2 infection (44, 45). In COVID-19 patients, the concentration of IP-10 was also positively correlated with disease severity, lung damage, and risk of death (46). Similar results were reported by de Morais Batista F. who found that IP-10 levels were significantly higher in patients with mild/severe symptoms of COVID-19 than in the control group (47). Elevated levels of IP-10 and MIG were also reported in other viral diseases, including hepatitis C (HCV) and infections caused by the human immunodeficiency virus (HIV) (48). The concentrations of IP-10 and MIG were positively correlated with viral loads and negatively correlated with the counts of CD4+ T cells (49, 50). In the present study, IP-10 and MIG levels were higher in stage 2 patients (pneumonia without respiratory failure) than in stage 1 (asymptomatic/mildly symptomatic) patients, and other authors also reported high concentrations of these chemokines in patients with moderate and severe COVID-19, which suggests that these chemokines can be useful biomarkers for monitoring the progression of the SARS-CoV-2 infection.

In the group of GFs evaluated in this study, basic-FGF and GM-CSF were the most useful markers for diagnosing COVID-19 and for differentiating between asymptomatic/mildly symptomatic patients (stage 1) and patients with pneumonia without respiratory failure (stage 2), which indicates that these parameters can be useful biomarkers for assessing disease severity. Basic-FGF is an 18-kDa polypeptide that belongs to the FGF family (51). It is found mainly in the basement membrane and subendothelial extracellular matrix of blood vessels (52). Basic- FGF induces the expression of genes involved in inflammatory processes, including proinflammatory cytokines (mainly IL-6) and their receptors, endothelial cell adhesion molecules, and prostaglandin pathway components (53). In the current study, basic-FGF levels were higher in patients with stage 2 COVID-19 than in asymptomatic/mildly symptomatic patients. In mild and moderate COVID-19, the increase in basic-FGF levels can be probably attributed to platelet, epithelial and endothelial cell dysfunctions caused by the SARS-CoV-2 virus because other researchers found that the levels of this GF were correlated with the levels of P-selectin (in the endothelium) and sCD40L in platelets (54–56). In moderate COVID-19, tissue repair processes can also trigger an increase in basic-FGF levels. Arsentieva et al. reported a decrease in basic-FGF levels in patients with acute COVID-19 (57), which could be attributed to the depletion of platelets, and epithelial and endothelial cells that are the main sources of basic-FGF (58, 59). In this group of patients, lower concentrations of basic-FGF can compromise wound healing because tissue regeneration is impaired by the cytokine storm (60). GM-CSF is produced at the inflammation site mainly by macrophages, T cells, fibroblasts, endothelial cells, epithelial cells, and cancer cells (61). This cytokine binds to the GM-CSF receptor (GM-CSF-R) which is composed of a ligand-specific alpha-chain (GM CSF-Rα) and beta-chain (GM CSF-Rβ) carrying the signal (62, 63). GM-CSF plays an immunomodulatory role by stimulating alveolar macrophages that scavenge microbes in the respiratory system (56). In healthy subjects, GM-CSF concentrations are low or undetectable, but this GF is overproduced during infection, including the SARS-CoV-2 infection (60), as demonstrated in the present study. In advanced stages of COVID-19, an overactive immune response results in excessive release of cytokines, including GM-CSF and IL-6 (62). Acute pneumonia is also observed in advanced stages of disease, which increases the risk of ARDS (64–66).The search for effective diagnostic biomarkers that support the differentiation of patients with different severity of COVID-19 has significant implications for clinical practice. Chemokines and GFs play an important role in the immune system which is activated in response to the SARS-CoV-2 infection. Research into chemokines and GFs can promote a better understanding of the mechanisms of disease, and it can contribute to the development of more effective treatment. This is the first study to compare chemokine and GF levels in asymptomatic/mildly symptomatic COVID-19 patients and patients with pneumonia without respiratory failure. The study demonstrated that pro-inflammatory cytokines are activated already in the early stages of COVID-19, which can increase the risk of severe infection and, in critical cases, can lead to death. In summary, the present findings indicate that MIG, basic-FGF, and GM-CSF can be considered as reliable parameters for differentiating between patients with different severity of the SARS-CoV-2 infection, and as predictive clinical indicators for monitoring the deterioration in the patients’ vital signs. The results of this study provide a useful diagnostic tool for monitoring patients with COVID-19, and they can also be helpful in selecting the appropriate treatment and reducing the risk of complications.

The study had several limitations. It was conducted on a small group of patients who were divided into two smaller groups based on their MEWS scores. Moreover, the participants were diagnosed with only stage 1 and stage 2 COVID-19, and further research should be carried out on a larger group of patients with all four stages of COVID-19. The presented results provide valuable preliminary insights for further clinical trials focusing on chemokines and growth factors and their diagnostic utility in a larger population of COVID-19 patients.

## Data availability statement

The original contributions presented in the study are included in the article/**Supplementary Material**. Further inquiries can be directed to the corresponding author.

## Ethics statement

The studies involving humans were approved by Medical University of Bialystok. The studies were conducted in accordance with the local legislation and institutional requirements. The participants provided their written informed consent to participate in this study.

## Author contributions

BW: Writing – original draft, Writing – review & editing. JD: Conceptualization, Data curation, Methodology, Writing – review & editing. MW: Investigation, Project administration, Supervision, Writing – review & editing. MŻ: Funding acquisition, Project administration, Supervision, Writing – review & editing. VD: Formal analysis, Validation, Investigation, Writing – review & editing. JM: Formal analysis, Validation, Supervision, Writing – review & editing. MM: Conceptualization, Investigation, Methodology, Writing – review & editing.
