# Supplementary material for: Changes in chemokine and growth factor levels may be useful biomarkers for monitoring disease severity in COVID-19 patients; a pilot study
Source: Front Immunol. 2024 Jan 4;14:1320362. doi: 10.3389/fimmu.2023.1320362 (PMC10794366; doi:10.3389/fimmu.2023.1320362)
Supplement: Supplementary file 1 [file Table_1.docx]

|  | **All patients with COVID-19** | **COVID-19 severity according to MEWS** | | **Control group** |
| --- | --- | --- | --- | --- |
| **Clinical parameters** |  | **1** | **2** |  |
| **Number of patients** | 100 | 53 (53%) | 47 (47%) | 50 |
| **Age (years)**  ≤55 56-75  >76 | 32 (32%)  31 (31%)  37 (37%) | 12 (20%)  15 (31%)  26 (49%) | 20 (41%)  16 (34%)  11 (25%) | 17 (34%)  25 (50%)  8 (16%) |
| **Sex**  Female Male | 65 (65%)  35 (35%) | 30 (65%)  23 (35%) | 35 (75%)  12 (25%) | 30 (60%)  20 (40%) |
| **Length of hospital stay (days)**  ≤10 10-20  >20 | 67 (67%)  22 (22%)  11 (11%) | 36 (69%)  13 (25%)  4 (6%) | 31 (66%)  8 (17%)  8 (17%) | -  - |
| **Comorbidities (n,%)**  Absent Present  Hypertension Diabetes mellitus Obesity  Coronary artery  disease  Other (e.g. cancers, hematological) | 43 (43%)  57 (57%)  22 (37%)  11 (17 %)  2 (4%)  15 (26%)  7 (10%) | 16 (31%)  29 (69%)  11 (41%)  5 (14%)  1 (3%)  9 (31%)  4 (11%) | 19 (42%)  28 (58%)  11 (41%)  6 (8%)  1 (8%)  6 (18%)  3 (3%) | 50 (100%) |
| Cough  Absent Present | 15 (15%)  85 (85%) | 1 (3%)  42 (97%) | 14 (22%)  43 (78%) | -  - |
| Fever  Absent Present | 63 (63%)  37 (37%) | 16 (20%)  37 (80%) | 0 (0%)  47 (100%) | -  - |

**Table S1**. Characteristics of the studied population
