# Supplementary material for: Changes in chemokine and growth factor levels may be useful biomarkers for monitoring disease severity in COVID-19 patients; a pilot study
Source: Front Immunol. 2024 Jan 4;14:1320362. doi: 10.3389/fimmu.2023.1320362 (PMC10794366; doi:10.3389/fimmu.2023.1320362)
Supplement: Supplementary file 2 [file Table_2.docx]

| **COVID-19 severity** | **1** | **2** | **p value** |
| --- | --- | --- | --- |
| **WBC x10^3^/mm^3^** | 6.63 (4.61-8.15) | 7.09 (5.65-8.94) | 0.0719 |
| **Neutrophils x10^3^/mm^3^** | 4.68 (3.02-6.68) | 5.12 (3.14-6.17) | 0.0458 |
| **Lymphocytes x10^3^mm^3^** | 1.15 (0.81-1.35) | 0.82 (0.41-1.13) | 0.2275 |
| **Monocytes x10^3^mm^3^** | 0.52 (0.28-0.67) | 0.61 (0.27-0.60) | 0.1955 |
| **Eosinophils x10^3^/mm^3^** | 0.034 (0.00-0.025) | 0.018 (0.000-0.010) | 0.4855 |
| **Basophils x10^3^/mm^3^** | 0.018 (0.010-0.020) | 0.016 (0.010-0.020) | 0.1567 |
| **RBC x10^6^/mm^3^** | 4.42 (4.01-4.86) | 4.22 (3.97-4.66) | 0.6029 |
| **PLT x10^3^/mm^3^** | 227.34 (160.50- 266.00) | 200.68 (147.000-248.000) | 0.0671 |
| **Creatinine mg/dl** | 0.978 (0.785-1.150) | 1.002 (0.780-1.130) | 0.7765 |
| **LDH U/I** | 420.26 (280.00-493.00) | 458.45 (323.000- 561.000) | 0.0412 |
| **INR** | 1.614 (1.080-1.300) | 1.796 (1.091-1.280) | 0.0854 |
| **Fibrinogen mg/dl** | 457.23 (322.00- 605.00) | 518.81 (385.00- 623.00) | 0.1458 |
| **D-dimers µg/l** | 3124.89 (605.50-1639.50) | 3897.28 (590.00- 1855.00) | p=0.3075 |

**Table S2**. A comparison of selected blood test results in COVID-19 patients with different MEWS scores
